# Risk factors for urinary incontinence during pregnancy among nulliparous women in the United Arab Emirates

**DOI:** 10.1097/MD.0000000000032738

**Published:** 2023-01-27

**Authors:** Hassan M Elbiss, Nawal Osman, Fikri M. Abu-Zidan

**Affiliations:** a Department of Obstetrics and Gyanecology, College of Medicine and Health Sciences United Arab Emirates University, Al-Ain, UAE; b The Research Office, College of Medicine, and Health Sciences United Arab Emirates University, Al-Ain, UAE.

**Keywords:** nulliparous, pregnancy, risk factors, urinary incontinence (UI)

## Abstract

Urinary incontinence (UI), which affects the quality of life, is associated with different risk factors during pregnancy. We aimed to study the risk factors related to UI during pregnancy among nulliparous women in the UAE. This is a prospective descriptive survey, which included all nulliparous women after the first 24 weeks’ gestation from 2012 to 2014 in a teaching hospital in the UAE. Participants were interviewed face-to-face, using a structured and pre-tested questionnaire and divided into 2 groups: those with UI and those without it. Factors which were statistically significant (*P* < .05) between the 2 groups were entered into an logistic regression backward logistic regression model to define the factors predicting UI. Five hundred one participants were interviewed. UI occurred in 106/501 (21.2%). The 2-sample comparison analysis showed that urinary tract infection (UTI) (47.2% vs 34.4%, *P* = .018) and its number of attacks (*P* = .007), chronic cough (28.3% vs 13.9%, *P* < .001) and chronic constipation (34.9% vs 19%, *P* < .001) were statistically significant between those who had UI and those who did not. The logistic regression backward logistic regression model showed that the risk factors which predicted UI were chronic constipation (*P* = .003), chronic cough (*P* = .008), and the number of UTI attacks (*P* = .036). UI affects one-fifth of nulliparous women in the UAE. Chronic cough, constipation, and repeated UTI infection, significantly increase the odds of UI during pregnancy. Addressing these risk factors may reduce the risk of UI.

## 1. Introduction

Urinary incontinence (UI) is one of several pelvic floor disorders that include pelvic organ prolapse, bowel incontinence, and other urinary and gastrointestinal tract abnormalities.^[1]^ UI is defined as involuntary leakage of urine when stressing, coughing, straining or laughing; or the inability to control urination on an urge.^[2]^ Stress incontinence, urge incontinence, and mixed incontinence are the 3 main types of UI.^[3]^ It is a pregnancy-related condition^[4]^ which is related to multiple anatomical and physiological factors including obesity,^[5]^ increased intra-abdominal pressure,^[6]^ hormonal changes,^[7]^ and multiparity.^[8,9]^ UI in pregnancy can affect the quality of women’s life, leading to psychological problems including anxiety, depression and low self-esteem.^[10]^ Furthermore, it may be associated with UI in later life.^[10–12]^

Forty to 50 percent of all mothers may have UI.^[11,12]^ Given the high incidence of diabetes in pregnancy, high birth weight, raised body mass index, and lack of pelvic floor muscles exercise awareness during and after delivery among Emirati women in the UAE, a high prevalence rate of UI can be expected compared with developed countries.^[13]^

UI in pregnancy may be under-reported because of the cultural belief that the condition is a normal part of carrying a baby inside the abdomen. This subject of UI in pregnancy has been infrequently evaluated in prospective studies in the region. In particular, there is a need to define risk factors for UI during pregnancy in the middle-eastern population so as to act on reducing them. We aimed to study the risk factors related to UI during pregnancy among nulliparous women in the UAE.

## 2. Patients and methods

### 2.1. Ethical considerations

Ethical approval was obtained from the Research and Ethics Committee of Al Ain Medical District in UAE (CRD 82/10, 12/01/2012). All mothers gave written informed consent to participate in the study.

### 2.2. Settings and participants

A prospective cohort study, recruiting all nulliparous women after 24 weeks’ gestation from 2012 to 2014 in a teaching hospital in the UAE. A questionnaire was used to collect data about women with symptoms of UI during pregnancy and postpartum among primigravid women. The inclusion and exclusion criteria were as follows. All consecutive nulliparous women after 24 weeks’ gestation presenting to the hospital were included in the study except those having any of the exclusion criteria which were: multiparous women, having UI before pregnancy, pregnant women with neurological disease (e.g., multiple sclerosis), or pregnant women with previous urogynaecological problems.

### 2.3. Questionnaire design and studied variables

The questionnaire developed in Arabic and English versions consisted of 5 parts: demography; medical and social history; UI during pregnancy; delivery and outcome; and postpartum course. The developed questionnaire was pre-tested and revised. The research interviewers were trained on the questionnaire and methods of interviewing the patients. The surface validity and content validity of the questionnaire depended on our previous research experience and publications in this area while piloting it and training of the interviewers was essential to assure its reliability.^[14–16]^ The questionnaire was field tested in a pilot study on female volunteers to ensure that the items and questions were acceptable and easy to understand. It was completed through face-to-face interviews with trained healthcare professionals. Socio-demographic characteristics included age, body mass index, nationality, occupation, and level of education. Pregnancy characteristics included urinary tract infection (UTI), chronic cough, chronic constipation, diabetes, and smoking. Additional labor and post-pregnancy characteristics were collected. This study adhered to the STROBE checklist.^[17]^

### 2.4. Statistical analysis

Assuming a true population prevalence of 40%,^[18]^ a sample size of around 500 nulliparous women has a 90% power, at a 2-sided *α* level of 5% (*P* < .05). The subjects of the study were divided into 2 groups, those with UI and those without it. Two sample statistical comparison was performed using the non-parametric methods of Fisher exact test for categorical data and Mann–Whitney *U* test for continuous data. Variables which had a loose *P* value of <.1 were entered into a backward logistic regression logistic regression model to define factors predicting UI. Data were analyzed using the Statistical Package for Social Sciences (IBM-SPSS version 26, Chicago, Il). A *P* value of <.05 was accepted as significant.

## 3. Results

There were a total of 501 participants. The baseline characteristics are shown in Table 1. UI prevalence was 106/501 (21.2%). The majority were in the age of 20 to 30 years, 74% were UAE nationals, 57% were housewives, only 30% had normal weight and >60% had higher education.

**Table 1:** Socio-demographic characteristics of participants of the study.

aCharacteristics		n	%
Age (yr)	<20	48	9.6%
	20–30	363	72.6%
	30–40	89	17.8%
	>40	0	0%
Nationality	National	371	74.1%
	Non-national	130	25.9%
Occupation cat	Housewife	288	57.5%
	Working	213	42.5%
BMI	Normal	144	29.9%
	Overweight	178	36.9%
	Obese	160	33.2%
Level of education	Illiterate	7	1.4%
	Elementary	52	10.4%
	High school	128	25.7%
	University/Higher education	312	62.5%

BMI = body mass index.

The 2 sample statistical comparison analysis comparing women with and without UI is presented in Table 2. Women with UTI (47.2% vs 34.4%, *P* 34.018), chronic cough (28.3% vs 13.9%, *P* 13.001), and chronic constipation (34.9% vs 19%, *P* 19.001) during the pregnancy had significantly more UI. The number of UTI infection attacks was significantly higher in those who had incontinence compared with those who did not (median [range] 1[1–7]) compared with 0 (0–0), *P* = .007, Mann–Whitney *U* test).

**Table 2:** Comparison between participants who had urinary incontinence (n = 106) and those who did not (n = 395).

	Urinary incontinence		*P* value
	Yes (n = 106)	No (n = 395)	
Age			.15
<20	5	43	
20–30	82	281	
30–40	19	70	
Level of education			.38
Illiterate	0	7	
Elementary	13	39	
High school	31	97	
University/Higher education	62	250	
BMI	28.6 (18–65)	27.6 (17–74)	.61
Nationality			.9
UAE	78	293	
Non UAE	28	102	
Occupation			.52
Housewife	57	232	
Working	49	164	
UTI during pregnancy			.018
Yes	50	136	
No	56	259	
Times of UTI	1 (1–7)	0 (0–0)	.007
Chronic cough			<.001
Yes	30	55	
No	76	340	
Chronic constipation			<.001
Yes	37	74	
No	69	320	
Diabetes			.17
No	86	342	
Yes	19	52	

BMI = body mass index, UTI = urinary tract infection.

*P value = Fisher exact test or Mann–Whitney test as appropriate.

Table 3 shows the backward logistic regression logistic regression model. It was highly significant (*P* < .001, Nagelkerke *R*^2^ = 0.07). The significant factors that predicted UI were chronic cough (*P* = .008), chronic constipation (0.003), and the number of UTI attacks (0.036).

**Table 3:** Backward LR logistic regression model evaluating risk factors for urinary incontinence during pregnancy among nulliparous women in the United Arab Emirates (n = 501).

Variable	Coefficient	SE	Wald	*P* value	Odds ratio	95% CI for odds ratio	
						Lower	Upper
Chronic cough	0.71	0.27	6.93	008	2.04	1.19	3.46
Chronic constipation	0.72	0.25	8.62	.003	2.06	1.27	3.34
Number of UTI attacks	0.26	0.12	4.39	.036	1.29	1.01	1.65
Constant	−1.8	0.16	127.7	<.001	0.17		

CI = confidence interval, LR = logistic regression, SE = standard error, UTI = urinary tract infection.

## 4. Discussion

Our study has shown that more than one-fifth of nulliparous pregnant women suffer from UI. Repeated UTI, chronic cough and chronic constipation are predictors of UI nulliparous pregnant women. Treating UTI, chronic cough, and chronic constipation may reduce the risk of UI during nulliparous pregnancy.^[19]^ A higher prevalence of UI during pregnancy has been previously reported ranging from 40 to 50% which is double the current study^[12,20]^ Similar to our findings, a study of the prevalence of UI among nulliparous women found an 18.3% rate.^[21]^ In comparison, non-pregnant women in Germany and Denmark had a prevalence rate of UI of 48.3 and 46.4% respectively.^[22]^

Despite the fact that UI is prevalent, two-thirds of women do not seek treatment.^[23]^ This is mostly due to social stigma, shame or embarrassment, and lack of awareness that this issue can be addressed.^[24]^ Factors predicting UI in pregnancy are reported to be occasional leakage during the non-pregnant period (less than once a month) and tobacco consumption.^[1,21]^ According to another recently published study from our setting, the prevalence of UI was nearly 50%, with stress incontinence being the most common type of UI.^[20]^ There are major differences between our study and this study.^[20]^ First, our study has more strict methodological criteria with properly calculated sample size. We studied 501 patients over 24 months compared with 105 patients studied over 3 months in the study by Syeda et al. Their small sample size may not reflect the real prevalence of UI. Although we have to consider the time difference of almost 10 years between the 2 studies. Furthermore, this is most properly the cause for having the non-significant comparisons for the risk factors in their study. Second, we studied only nulliparous women compared with Syeda et al who studied all pregnant women. Third, we collected all our data prospectively while they collected the associated risk factors retrospectively from the files which has a much higher risk of missing data. Finally, Syeda et al did not describe the methods of their 2-sample statistical comparisons compared with the rigid statistical methods of our study.

UI before and during pregnancy was associated with postpartum stress UI.^[25]^ Our study has shown that chronic cough, chronic constipation, and repeated UTI are associated with UI. Accordingly, women and their carers should be educated about the associated factors during their pregnancy. Chronic cough and constipation should be treated immediately to avoid the undue aggravation of the already stressed pelvic floor muscles. Additionally, pelvic floor muscle exercises should be advised for strengthening purposes in particular because UI during pregnancy has a strong relationship with the development of UI in later stages of life.^[26]^

## 5. Limitations of the study

We have to acknowledge that our study has certain limitations. First, This study stemmed from a single hospital from UAE which may not be generalizable to the whole country. Second, we included only nulliparous women which also limits its generalizability to all pregnant women. Third, we used a survey as our research tool. Surveys have certain limitations. They depend mainly on the participants’ responses which may be subjective, affected by recall bias, or the patient may feel embarrassed to complain about UI which underestimates the real prevalence of UI. Fourth, the data is nearly ten years old. Despite that, the message of our study is still relevant and important because UI can affect the quality of life of pregnant women. Furthermore, the prospective nature of the study and its large sample size strengthen it. Finally, the *R*^2^ of the logistic model was very low (0.07) indicating that, although the model was highly significant, it explains only 7% of the variation in the data and that there are other important factors which were not included in the model. Despite these limitations, we think that our study is important and that it addresses an important area. It has accepted reliability and validity depending on our previous publication and expertise in this area, and finally, we used trained interviewers for face-to-face data collection.

## 6. Conclusions

UI affects one-fifth of nulliparous women in the UAE. Chronic cough, constipation, and repeated UTI infection, significantly increase the odds of UI during pregnancy. Addressing these risk factors may reduce the risk of UI.

## Author contributions

**Conceptualization:** Hassan Elbiss, Nawal Osman.

**Data curation:** Hassan M. Elbiss, Nawal Osman.

**Formal analysis:** Hassan M. Elbiss, Nawal Osman, Fikri M. Abu-Zidan.

**Methodology:** Hassan M. Elbiss, Fikri M. Abu-Zidan.

**Software:** Nawal Osman, Fikri M. Abu-Zidan.

**Supervision:** Hassan M. Elbiss, Fikri M. Abu-Zidan.

**Validation:** Hassan M. Elbiss, Fikri M. Abu-Zidan.

**Visualization:** Hassan M. Elbiss.

**Writing – original draft:** Hassan M. Elbiss, Nawal Osman, Fikri M. Abu-Zidan.

**Writing – review & editing:** Fikri M. Abu-Zidan.
